# Chest Wall Deformity and Rib Fractures in Breast Cancer Patients Following Reconstruction with Tissue Expanders

**DOI:** 10.3390/medsci14060676

**Published:** 2026-10-15

**Authors:** Serghei Covantsev, Anna Sukhotko, Aziz Zikiryakhodzhaev, Maria Potapova, Ilona Duadze, Zakhar Kovalenko, Alexandru Corlateanu

**Affiliations:** 1 Department of Oncology, National Medical Research Treatment and Rehabilitation Centre of the Ministry of Health of Russia, Moscow 125367, Russia; 2 Department of Oncology and Reconstructive Plastic Surgery of the Breast and Skin, P.A. Hertzen Moscow Oncology Research Institute, Moscow 125284, Russia; 3 Department of Oncology, Sechenov First Moscow State Medical University ( Sechenov University), Moscow 119991, Russia; 4 Department of Oncology, Peoples’ Friendship University of Russia, Moscow 117198, Russia; 5 Department of Respiratory Medicine, Nicolae Testemițanu State University of Medicine and Pharmacy, MD-2025 Chisinau, Moldova; alexandru.corlateanu@usmf.md

**Keywords:** breast cancer, tissue expanders, breast implants, chest wall, rib fracture

## Abstract

Breast cancer is the most frequent type of cancer in women and accounts for 11.6% of new cancer cases worldwide and 6.9% of cancer-related deaths. Breast surgery has evolved over time from radical mastectomy toward a preference for breast-conserving surgery and the widespread use of oncoplastic surgery. However, a subgroup of patients still undergo radical mastectomies with subsequent reconstruction via tissue expanders and breast implants. However, this subgroup of patients frequently exhibit mild-to-moderate chest wall deformities and, in rare instances, rib fractures, and the long-term effects of tissue expanders on chest wall deformity and rib fractures require further evaluation. The underlying cause is most likely a combination of metabolic changes due to systemic and hormonal treatment, radiotherapy, and local compression of the chest wall. Although imaging abnormalities appear relatively common in some studies, symptomatic complications and true rib fractures are uncommon and supported mainly by small-series and case reports. Further evaluation is required to determine if a subset of patients require treatment for this condition.

## 1. Introduction

Breast cancer is the most frequent type of cancer in women and accounts for 11.6% of new cancer cases worldwide and 6.9% of cancer-related deaths [1]. Based on the current guidelines, the management of breast cancer patients includes surgery, radiation therapy, chemotherapy, targeted therapy, and hormonal therapy [2,3]. Therefore, optimal management requires a multidisciplinary team of healthcare providers.

Breast surgery has evolved over time from radical mastectomy toward a preference for breast-conserving surgery and the widespread use of oncoplastic surgery. However, a subgroup of patients still undergo radical mastectomies with subsequent reconstruction via tissue expanders and breast implants [4].

Tissue expanders are temporary balloon-like devices used in breast reconstruction after a mastectomy. The expander is placed under the chest skin and/or muscles and slowly stretches the tissue over several months. This creates a pocket in which to hold a permanent breast implant [5]. However, tissue expanders create significant pressure on the chest wall, which can lead to deformity, trauma, and even respiratory complications.

The degree of bony deformation is most likely caused by a combination of osteoporosis, chronic corticosteroid (CS) use, hormone therapy, chemotherapy, comorbidities, and radiation therapy.

This review aims to analyze chest wall deformities in breast cancer patients following reconstruction with tissue expanders.

## 2. Search Methodology

SC and MP performed a literature search using the terms “tissue expander,” “breast cancer,” “chest wall,” “deformity,” “rib fracture,” and “breast reconstruction,” along with the MESH terms in English, with no year limitation, in May 2026. The reference list of the articles was carefully reviewed as a potential source of information. The search was based on Medline, Scopus, and Google Scholar. A total of 164 publications were initially selected and screened. Papers were excluded if the article was out of the scope of the review, the full text was unavailable, or the article was duplicated. The 70 publications selected were analyzed, and their synthesis was used to write the review and analyze the role of tissue expanders in chest wall deformity, rib fracture, and breast reconstruction.

## 3. Rib Fractures

Rib fractures account for 7–10% of all fractures, with an annual incidence of 18–29 per 100,000 person-years [6,7,8,9]. Rib fracture-associated mortality is 4.0–6.9% and depends on age, Injury Severity Score (ISS), and comorbidities [9,10]. Among those with a rib fracture, the incidence of subsequent fractures is 10.2/100 person-years. In this case, the risk of mortality increases by 4.9-fold (95% CI 2.0 to 11.8) in women within the first year post-fracture [8].

Spontaneous rib fractures after treatment for primary breast cancer are not uncommon. Although metastatic disease accounts for approximately 30% of spontaneous rib fractures and should constitute the first line of diagnostic investigation, other conditions may also lead to decreased bone mineral density, including primary or secondary osteoporosis, chemotherapy-induced menopause, aromatase inhibitors, radiation therapy, and long-term bisphosphonate use [11]. Rib cage compression by tissue expanders is another factor that is not commonly studied, with only a few cases reported in the literature.

## 4. Bone Mineral Density

Bone mineral density and age are among the key factors associated with rib fracture [12]. However, there are multiple risk factors, such as calcium intake, vitamin D deficiency, smoking, current physical activity, and muscle status [13,14].

Interestingly, BMD is tightly linked to a number of comorbidities, such as cardiovascular disease, respiratory conditions, autoimmune disease, and others, suggesting that bone tissue metabolism is easily affected by both internal and external factors [15,16,17].

Osteoporosis is significantly associated with higher rib fracture rates. Furthermore, both osteopenia and osteoporosis patients tend to have a significantly higher number of fractured ribs and fracture sites on their ribs [18].

## 5. Corticosteroid Use

Systemic corticosteroids are often used during chemotherapy due to their anti-inflammatory properties, and to reduce the side effects of cytotoxic drugs such as vomiting, allergies, edema, and pain [19]. CS use for 2–6 weeks decreases whole-bone mineral density and the z-score by 1.82–11.42% [20]. A meta-analysis of 42,542 men and women followed for 176,286 person-years found that those treated with CS had a higher risk of fracture than those with no history of CS use. The relative risk at 50 years of age was 1.98 and the relative risk at 85 years of age was 1.66 compared to a control group who never received CS [21]. Spontaneous rib fractures are frequently seen in patients who receive long-term systemic CS for other conditions [22].

## 6. Chemotherapy, Aromatase Inhibitors, and Bone Mineral Density

Chemotherapy affects gonadal hormone production, which increases bone resorption and decreases BMD. BMD loss in patients receiving chemotherapy occurs more rapidly and to a greater degree than normal age-related osteoporosis, increasing the risk for fracture [23]. Patients who receive aromatase inhibitors (AIs) are associated with a higher risk of osteoporosis development in comparison to tamoxifen (TMX) or chemotherapy. Notably, AIs administered subsequent to a combination of chemotherapy and anti-HER2 therapy exhibited the highest risk of osteoporosis development [24].

The introduction of aromatase inhibitors (AIs) has opened new possibilities in the successful treatment of ER-positive breast cancer. They are currently the standard of treatment in the adjuvant therapy of post-menopausal women with hormone-responsive breast cancer in upfront, switch, and sequential treatment settings [25,26].

However, anastrozole is associated with accelerated bone loss over the 5-year treatment period. Among anastrozole-treated patients, there was a decrease in median BMD from baseline to 5 years in lumbar spine (−6.08%) and total hip (−7.24%) compared with the tamoxifen group (lumbar spine, +2.77%; total hip, +0.74%). Patients with pre-existing osteopenia are likely to require monitoring and bone-protection strategies. Patients with normal BMD would not appear to require monitoring beyond the recommendation for healthy post-menopausal women [27].

Oral bisphosphonates help to maintain BMD but are still associated with a persistent fracture risk, particularly in older women [28]. On average, after 2 years, osteopenic patients treated with ibandronate gained +2.98% (range −8.9, +19.9) and +0.60% (range −9.0, +6.9) at the lumbar spine and hip, respectively. Patients treated with placebo, however, lost −3.22% (range −16.0, +4.3) at the lumbar spine and −3.90% (range −12.3, +7.2) at the hip [29]. The addition of oral risedronate in post-menopausal breast cancer patients receiving anastrozole has a favorable effect on BMD. Patients with pre-treatment osteopenic-to-osteoporotic status should be treated with a combination of both therapies in order to avoid bone loss induced by aromatase inhibition [29,30].

## 7. Radiation Therapy

Radiation-induced rib fractures refer to the occurrence of rib fractures following radiotherapy to the region close to a rib or within the path of a rib. They are considered pathological fractures as they happen in the underlying abnormal bone affected by radiation therapy. Conventional radiotherapy, stereotactic body radiotherapy, and brachytherapy aim to minimize damage to healthy tissues near the cancer site, but still pose a potential threat of spontaneous rib fractures [31].

Radiation therapy to the breast or chest wall has long been implicated as a major contributor to rib fractures. In 1988, Overgaard reported rib fractures in 6–19% of patients who had undergone radiation therapy; however, a higher rate was observed in patients who received a larger dose per radiation fraction [32]. Changes in radiation therapy protocols and technical advancements led to a decrease in rib fracture incidence, which is indicated in some guidelines to be less than 2% [33]. However, the rates of radiation-induced rib fractures in multiple studies is approximately 4–5% [34,35,36]. Hypo-fractionated RT may result in fewer complications as symptomatic rib fractures were 0.1% in START A and 0.3% in START B trials [37,38].

Nevertheless, in a retrospective review of 867 patients with breast cancer who underwent surgery and radiation therapy and were followed-up with at least three bone scans (every 12 months), 159 (18%) developed rib fracture. Among the 159 cases, 127 (80%) occurred in the ipsilateral breast and 32 (20%) in the contralateral breast. Abnormal bone density, chemotherapy, and radiation therapy were significant risk factors for rib fracture. Furthermore, multivariate analysis of radiotherapy subgroups showed that hypofractionated radiotherapy increased the rate of spontaneous rib fracture (*p* = 0.002) [39].

In dosimetric analysis, near-maximum rib dose (D2cc) best predicted ipsilateral rib fracture. In a multivariate analysis including dosimetric variables, D2cc EQD2 ≥ 52 Gy was the only significant risk factor for ipsilateral spontaneous rib fracture [40].

Another possible risk factor is limited distance from the rib to the tumor (<2.0 cm). Among the dosimetric parameters used for receiver operating characteristic analysis, the maximum dose showed the highest area under the curve. The 3-year estimated risk of RIRF and the determined cutoff value were 45.8% vs. 1.4% (maximum dose, ≥42.4 Gy or less), 51.6% vs. 2.0% (V40, ≥0.29 cm(3) or less), 45.8% vs. 2.2% (V30, ≥1.35 cm(3) or less), 42.0% vs. 8.5% (V20, ≥3.62 cm(3) or less), or 25.9% vs. 10.5% (V10, ≥5.03 cm(3) or less) [41].

Most of the patients with SRF (87.3%) were asymptomatic, and the remaining symptomatic patients complained of mild tenderness or chest wall discomfort; these symptoms all resolved within 6 months without any treatment [40].

## 8. Tissue Expander, Rib Cage Deformity, and Rib Fracture

The use of breast implants in breast reconstruction surgery began in the early 1960s with silicone gel-filled implants. Radovan subsequently introduced the concept of breast tissue expansion in the early 1980s [42]. Although breast reconstruction is a frequent procedure, up 23.1% of patients develop complications and 40.2% of patients require elective revisions [43].

Contemporary breast reconstruction is characterized by a flexible number of techniques for individual anatomy, cancer therapy plans, and patient preferences. The choice of expander position, the expansion strategy, and the use (or avoidance) of radiotherapy all influence short-term recovery and long-term results. A two-stage breast reconstruction is a common approach in many settings, where the tissue expander is subsequently changed to an implant. Expanders are placed during the mastectomy stage or afterwards and gradually filled to create a pocket; this is then followed by exchange with a definitive implant. This allows for gradual tissue stretching and control over the final shape. There are several common planes suitable for expander placement. The submuscular plane is where the expander sits partially under the pectoralis major muscle. The muscle coverage leads to less visible rippling in thin skin but usually more post-operative pain, slower initial expansion, and possible animation deformity. In the dual-plane, partial muscle coverage with lower pole release allows the implant to sit fully in the pocket with improved coverage. This usually allows for better control of the upper pole contour but still involves muscle coverage; therefore, some animation or discomfort may persist. Prepectoral expander placement on top of the chest wall, beneath the skin and subcutaneous tissue, is associated with no muscle-related animation, often less post-operative pain, and faster initial recovery; however, there is greater concern with respect to skin flap viability and rippling, and it requires careful patient selection [42,43,44,45].

Chest wall deformity after tissue expansion for breast reconstruction is a frequent but understudied topic. Rib and chest wall contours change under 69–81% of the expanders. Chest CT can help to evaluate rib chest changes, while routine chest roentgenograms are not a sensitive method for evaluating these deformities. After removing the expanders and placing permanent implants along with capsulotomy, the mean deformity index decreased by 57%% after 10.5 months [44]. Overinflation and maximum tissue expansion seem to be among the risk factors [45].

A prospective longitudinal study of the chest wall in 36 patients who underwent immediate two-stage expander-to-implant surgery was conducted to evaluate the possible causes of chest wall deformity. Computed tomography (CT) scans of the chest walls were taken before the second-stage reconstruction and after 1 year. The study demonstrated that 22.22% of patients had no deformities, 38.88% had a flattening rib cage profile, 27.78% had inflection of the profile rib with pleural sinking, 11.11% had infraction, and none had rib fractures. At the 1-year follow-up after the tissue expander-to-implant change, 61.11% of patients had the same degree of chest wall deformity, 27.77% showed an improvement. and 11.11% had a higher-grade deformity [46].

The pre- and post-operative difference in anteroposterior chest length, on average, decreases by 4.16 mm (range: −2.16 to 13.82 mm). In a multivariate analysis, capsular contracture and age were the independent prognostic factors correlated with these changes [47]. Capsular contracture can induce higher pressure, and it has a positive correlation with deformity when the expander is in an overinflated state [48].

A comparison of the studies that demonstrate chest wall deformities is given in Table 1.

In a 6-year retrospective review of tissue expander use for breast reconstruction, 2.2% (2 cases out of 91) patients had rib fractures, particularly in the subset of irradiated breast reconstruction patients [50]. Based on the available data (Table 2), it seems that tissue expander-induced rib fractures are more common in irradiated and overinflated expanders. Therefore, it seems that overinflation is one of the possible risk factors for chest wall deformity and fractures. The current data suggests that inflation of 500 cc and more might be one of the predisposing risk factors, although this evidence is mostly based on case reports (Table 2).

**Figure 1:**
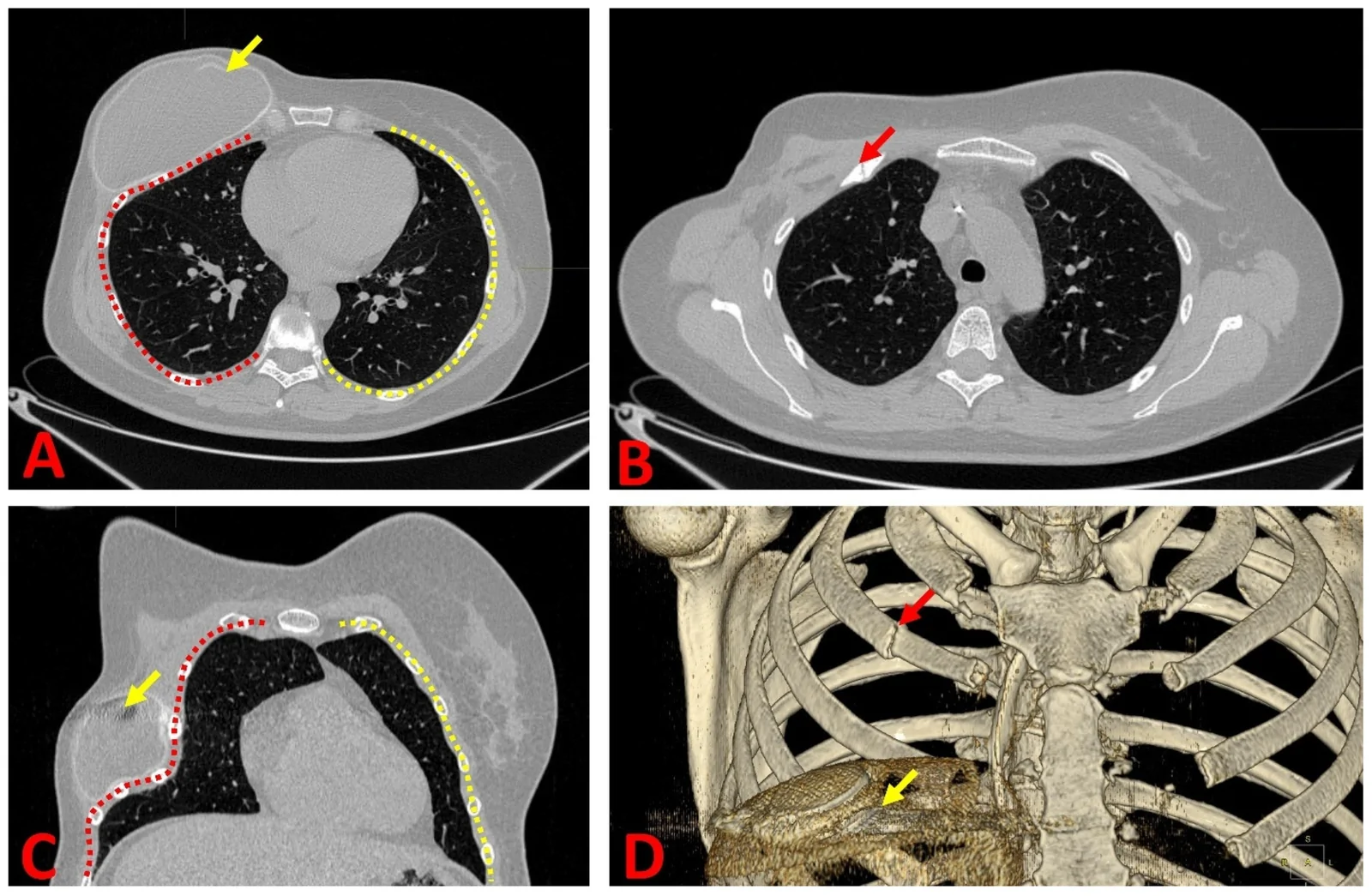
(**A**) Tissue expander-induced chest wall compression with rib flattening and second rib fracture (axial plane, red dotted line—right chest wall boundaries; yellow dotted line—left chest wall boundaries; yellow arrow—tissue expander). (**B**) Second rib fracture (red arrow indicates the fracture zone). (**C**) Tissue expander-induced chest wall compression with rib cage inflection and pleural sinking (axial plane, red dotted line—right chest wall boundaries; yellow dotted line—left chest wall boundaries; yellow arrow—tissue expander). (**D**) 3D reconstruction of the chest wall (red arrow indicated fracture zone, yellow arrow indicated tissue expander).

**Figure 2:**
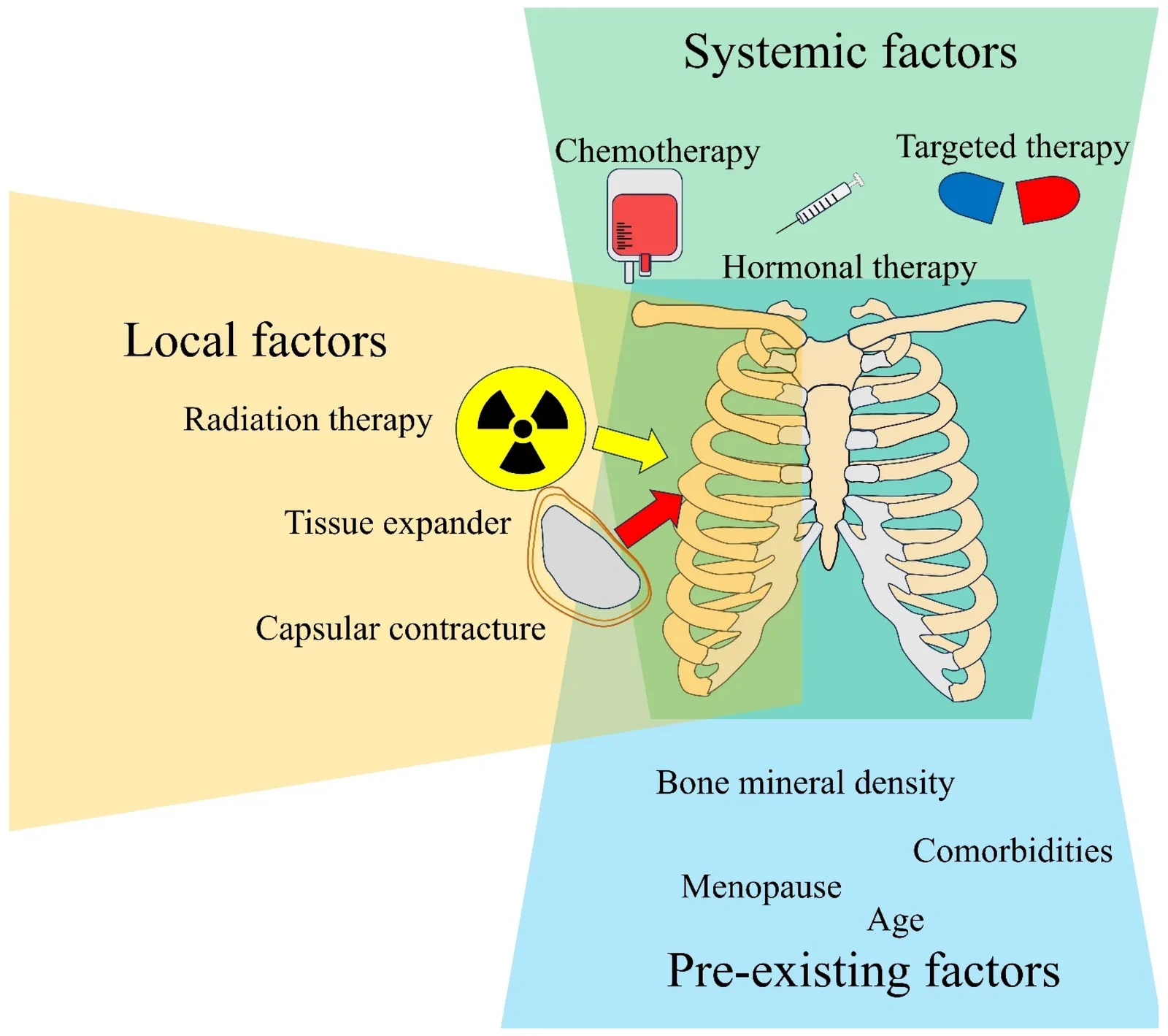
Schematic representation of the main systemic, pre-existing, and local factors that lead to chest deformity and rib fracture in patients undergoing breast reconstruction.

The available data demonstrates that chest wall deformity is common during breast reconstruction with tissue expanders and leads to progressive compression, flattening, inflection, and pleural sinking (Figure 1). In incidental cases, it can cause rib fractures, especially in patients with pre-existing factors in combination with systemic and local factors (Figure 2). The key pre-existing factors are those related to bone mineral density and are age, female sex, comorbidities, and estrogen status. The combination of these factors leads to osteomalacia and osteoporosis and does not allow the rib cage to resist pressure. The combination of systemic external factors includes the combination of medical treatments that the patient had or is currently undergoing (endocrine, chemotherapy, targeted therapy) that are all linked to bone loss. Finally, a number of local factors, such as radiation therapy and expander placement, are also relevant. Radiation therapy is linked to local bone destruction and possible fractures. Tissue expander expansion causes increased pressure on the chest wall, which can lead to its changing and, in rare instances, rib fracture. When this is combined with capsular contracture, the pressure on the rib cage can increase even more. After tissue expansion, minor chest wall deformation is common, but more serious moderate-to-severe deformities can cause significant morbidity, albeit rarely.

## 9. Respiratory Function in Breast Cancer Patients Undergoing Complex Treatment

Only 0.67–5.26% of patients who undergo two-stage breast reconstruction experience a clinically noticeable dyspnea most likely due to post-expansion chest compression [44,46]. Overall, it seems unlikely that respiratory deviations can occur due to tissue expanders (except in cases of overinflation); other causes may therefore play a role.

There seems to be a decrease in vital capacity (VC) and inspiratory capacity (IC). Respiratory muscle strength reduced 48 h after surgery but returned to baseline after 30 days and was maintained 60 days after the surgery. Therefore, it seems that breast surgery itself has only temporary effects of respiratory function [53]. Dyspnea following breast surgery seems to be incidental, and there are no significant differences in spirometry analysis changes from before the first surgery (mastectomy with tissue expander insertion) to after the second surgery (tissue expander changed to implant) [46].

It seems that systemic chemotherapy and local radiation therapy are the main factors underlying respiratory changes in breast cancer patients. Systemic chemotherapy for breast cancer appears to decrease forced vital capacity (FVC), forced expiratory volume in 1s (FEV1), and the forced expiratory flow between 25% and 75% of vital capacity (FEF25–75%) [54]. A significant drop in FVC, FEV1, and DLCO was noticed on day 90 after the completion of locoregional post-mastectomy radiotherapy, while MMEF25–75 and VO2max were not significantly affected (*p*-values 0.075 and 0.062, respectively), favoring a restrictive lung injury pattern but with no clinical symptoms [55]. However, this may depend on the chemotherapy regiment. Neoadjuvant chemotherapy for breast cancer can reduce the carbon monoxide diffusing capacity (DLCO) and percentage DLCO predicted value (DLCO%) (*p* < 0.001), increasing maximal ventilatory volume (MVV) (*p* = 0.004) and leading to dyspnea (*p* = 0.031) and fatigue (*p* < 0.001) [56]. Lung toxicity is also seen in targeted therapies such as drug-conjugated antibodies, PI3k-AKT-m-TOR inhibitors, CDK4/6i, and immunotherapy [57].

Respiratory changes are relatively frequent in patients during systemic chemotherapy and radiation therapy. Therefore, patients who have undergone breast reconstruction with expanders are more likely to have a pre-existing respiratory deviation that might be only slightly influenced by chest wall compression.

## 10. Risk Assessment and Monitoring

There are a number of risk factors that lead to fractures in patients with breast cancer. Absolute fracture risk can be calculated using the FRAX tool. FRAX is a computer-based algorithm that estimates the 10-year probability of hip fracture and major osteoporotic fracture (which includes the hip, clinical spine, distal forearm, and humerus fractures) [58]. The FRAX score has been incorporated into many guidelines to determine the patients for whom pharmacologic therapy should be initiated [58].

Patients at all risk levels should undergo lifestyle interventions such as adequate calcium and vitamin D intake, weight-bearing and muscle-strengthening exercises, fall prevention strategies (vision correction, home hazard removal), smoking cessation, and limiting alcohol consumption [59,60,61]. For high-risk patients undergoing endocrine therapy, confirmed osteoporosis pharmacological therapy, e.g., antiresorptive medications (bisphosphonates or denosumab), is recommended to slow bone breakdown. For very-high-risk patients who have suffered multiple fractures, anabolic drugs (e.g., teriparatide, romosozumab) may be used to actively build new bone [59,60,61]. It should be noted, however, that long-term bisphosphonates use may actually increase the risk for low-energy fractures due to suppressed bone turnover [62,63].

The management of comorbidities is another important goal as they can lead to fractures and poor healing [64].

Chest wall deformities can be evaluated via chest CT scans as patients with breast cancer undergo evaluation for recurrence and lung metastasis or during other clinical procedures [65]. The chest wall can be evaluated for rib infraction, fracture, and deformity, although these scores will require validation (Table 3 and Table 4) [44,45].

## 11. Rib Fracture Management

Isolated rib fractures can be managed with conservative therapy (analgesia, rest). Rib taping is no longer the recommended treatment as it can lead to increased inspiratory effort [66]. Supplementary oxygen is usually sufficient to treat hypoxia. Patients developing more significant hypoxia or hypercarbia may benefit from high-flow nasal cannulae or non-invasive ventilation [67].

The severity of rib fractures can be determined based on the number of ribs broken, unilateral or bilateral fracture, the position on the rib (anterior, lateral, or posterior), displacement, and the presence or absence of a flail segment [67].

In cases of rib fracture nonunion, chest wall deformity or defects, refractory rib fracture, pain-causing respiratory failure, and flail chest surgical stabilization are indicated [68]. Emerging evidence suggests that these should also be considered in cases of multiple fractures, particularly in fractures 4–9 [67].

There is evidence to suggest that if surgery is delayed beyond 3 days after the injury, then ICU and hospital stay durations are disproportionately prolonged and there is little benefit to surgical fixation beyond 10 days post-injury because of the high likelihood of suitable patients having already developed pneumonia [67,69].

Any other underlying injuries, such as pneumothorax or hemothorax, should be appropriately managed via the insertion of a chest tube if indicated [66]. Particular attention needs to be paid to lung, liver, and splenic injury; however, these are more common in trauma patients [70].

There are no universally accepted assessment tools for patients with rib fractures, and those that have been developed (rib fracture score, chest trauma score, and RibScore) are associated with traumatic rib fractures [67].

Several important points can be drawn from cases of traumatic rib fractures that require surgery. Stabilization of rib fractures is necessary in cases where there is a likelihood of respiratory compromise (symptomatic fractures of three or more consecutive ribs, flail segment, significant comorbidities); difficult pain management in non-intubated patients despite appropriate use of analgesic drugs; intubated patients in whom weaning from artificial ventilation fails; thoracotomy being undertaken for associated thoracic injury; and anterolateral flail chest or severe chest wall deformity with or without pulmonary herniation [67]. As radiation-induced rib fractures and expander-induced rib fractures tend to lead to isolated rib fractures, it is safe to presume that they usually do not require surgical management.

## 12. Current Research, Future Directions, and Limitations

Current research indicates that chest wall deformity is a relatively common, multifactorial issue in breast reconstruction via a tissue expander, influenced by expansion strategy, radiotherapy, and patient bone mineral density. While some deformities improve after final implant placement, risks such as rib fractures exist, especially with irradiation and overinflation. There is a clear need for larger, standardized studies that integrate expansion protocols, surgical techniques, bone health, chemotherapy, and RT into the analysis. The current review has certain limitations: It is based on limited data, most of which indicates that chest wall abnormalities in patients with two-staged breast reconstruction are relatively common, while rib fractures are seen in incidental cases. Both chest wall deformities and rib fractures in these patients did not lead to respiratory complications; however, this requires further evaluation.

## 13. Conclusions

Tissue expanders are frequently used for two-stage breast reconstruction when the patient needs to undergo mastectomy as the initial surgical treatment. This group of patients frequently have mild-to-moderate chest wall deformities and, in rare instances, rib fractures. The underlying cause is most likely a combination of metabolic changes due to systemic and hormonal treatment, radiotherapy, and local compression of the chest wall. Although imaging abnormalities appear relatively common in some studies, symptomatic complications and true rib fractures are uncommon and supported mainly by small-series and case reports. Further evaluation is required to determine if a subset of patients require treatment of the condition.

## Figures and Tables

**Table 1:** Studies that demonstrate chest wall deformity during breast reconstruction with tissue expanders.

Author, Year, Reference	Cases	Results	Clinical Implications
Dickson and Sharpe, 1987 [49]	75 patients	Two patients (2.66%) had rib cage deformity.	It is necessary to use larger implants for reconstruction due to rib depression.
Sinow et al., 1991 [44]	19 patients	A total of 94% of expanders had some thoracic abnormality after tissue expansion. Rib and chest wall contour changes were observed under 81 and 68% of the expanders, respectively.	Only one patient experienced a clinically noticeable complication from chest compression—namely, transient post-expansion exertional dyspnea.
Moor et al., 1996 [45]	150 patients	A total of 53% had some chest wall abnormality.	Chest wall deformities were more statistically significant in the delayed reconstruction group than in the immediate group.
Cherubino et al., 2016 [46]	36 patients	A total of 22.22% of patients had no deformities, 38.88% had a flattening rib cage profile, 27.78% has inflection of the profile rib with pleural sinking, 11.11% had infraction, and none had rib fractures. At the 1-year follow-up after the tissue expander-to-implant change, 61.11% of patients had the same degree of chest wall deformity, 27.77% showed an improvement, and 11.11% had a higher-grade deformity.	Chest wall deformities commonly occur after tissue expander/implant reconstruction. However, the size of the expander, reconstruction timing and filling volume are not correlated with deformity development.
Makiguchi et al., 2019 [48]	34 patients	The post-surgical chest wall deformity index was significantly higher than the pre-surgical chest wall deformity index (3.66 ± 3.23% vs. 0.03 ± 2.74%, *p* < 0.001), showing that chest wall deformity occurred after maximum expansion. In a multiple linear regression model, capsular contracture emerged as a significant predictor of increased post–pre chest wall deformity index (*p* = 0.003).	Chest wall deformity occurred after maximum expansion, but no rib fracture or dyspnea occurred in any patients.
Kim et al., 2022 [47]	57 patients	The pre- and post-operative difference in anteroposterior length was statistically significant. The average depth of chest wall deformity was 4.16 mm (range: −2.16 to 13.82 mm).	Capsular contracture and age were the independent prognostic factors correlated with chest wall deformity.

**Table 2:** Cases of expander-induced rib fractures.

Author, Year, Reference	Case	Treatment	Possible Risk Factors
McKinney et al., 1987 [51]	A 53-year-old female with a 550 cc expander with multiple rib fractures.	Tamoxifen, 5FU, melphalan, prednisone	Severe osteoporosis, chronic steroid use, and peripheral vascular disease
Tseng et al., 2010 [50]	A 46-year-old woman with a higher fill volume of the left tissue expander (430 mL) compared with the right tissue expander (260 mL). Chest CT revealed a healing anterolateral fifth rib fracture and a concave chest wall deformity deep to the implant. A 55-year-old woman who developed sudden onset of chest pain under her left breast tissue expander with associated mild skin erythema during the expansion process. Intraoperatively, there was a mobile rib segment. A post-operative chest computed tomographic scan confirmed the presence of a left fifth rib fracture and a moth-eaten appearance to the surrounding left ribs, consistent with post-irradiation skeletal changes.	Radiotherapy	Radiotherapy
Tirone et al., 2010 [52]	A 45-year-old woman whose 500 cc tissue expander overinflated to 600 cc. She experienced chest pain under her left breast tissue expander after a sneeze.	Not specified	Irradiation

**Table 3:** Chest wall deformity scores.

Category	Points
Ribs	
-Normal	0
-Flattening	1
-Moderate displacement	2
-Severe displacement	3
-Fracture	4
Soft tissue	
-Normal	0
-Protrusion beyond pleura	1
Chest wall contours	
-Normal	0
-Mild	1
-Moderate	2
-Severe	3

**Table 4:** Chest wall deformity stages.

Degree of Severity	Total Points
A. Normal	0
B. Mild	1–4
C. Moderate	5–6
D. Severe	7–9

## Data Availability

No new data were created or analyzed in this study. Data sharing is not applicable.
